# Predictors of Play Development at Home After Parent-Mediated Early Intervention for Autistic Preschool Children

**DOI:** 10.3390/bs16030387

**Published:** 2026-03-08

**Authors:** Ya-Chih Chang, Wendy Shih, Connie Kasari

**Affiliations:** 1 Department of Special Education and Counseling, California State University, Los Angeles, CA 90032, USA; 2 Department of Psychiatry and Behavioral Sciences, University of California, Los Angeles, CA 90095, USA; wshih@mednet.ucla.edu (W.S.); kasari@gseis.ucla.edu (C.K.)

**Keywords:** parent-mediated intervention, play skills, predictors, JASPER

## Abstract

Play is a critical developmental domain linked to social communication, cognitive growth, and later peer relationships; however, young autistic children often demonstrate delays in their play skills, especially higher level play skills (i.e., symbolic play). Although play-based, parent-mediated interventions show promise in improving parent strategies for engaging their children in play, we know less about how these strategies translate to child play improvement outside of the therapeutic sessions. The current study examined the home activities of 97 caregiver–child dyads following their participation in parent-mediated Joint Attention Symbolic Play Engagement Regulation (JASPER), among families in low socioeconomic circumstances. Naturalistic home observations identified the types of daily activities in which the dyads engaged, including play. Within play contexts, children whose caregivers received JASPER demonstrated a greater change from functional to symbolic play compared to those in a parent education control condition. Additionally, child-level, but not family-level, characteristics predicted greater gains in symbolic play. The results provide insight into the ecological validity of parent-mediated, play-based interventions delivered in home settings and highlight factors associated with variability in play outcomes. These findings have implications for tailoring parent-mediated interventions to better support equitable and sustainable developmental gains for autistic children.

## 1. Introduction

Play during early childhood is widely recognized as a foundational context for learning, communication, and social development. Engagement in varied forms of play supports the emergence of cognitive skills, symbolic thinking, and the development of social communication skills (Chang et al., 2024; Lifter et al., 2011; Mundy et al., 1987). In typically developing children, play progresses from functional, object-based actions to increasingly complex symbolic and pretend play. Symbolic play may involve using objects to represent something else or adopting the role of a character, for example, pretending a block is a phone or acting as a superhero. These behaviors reflect children’s growing representational abilities and are closely associated with later social competence and language development (Freeman et al., 2015; Sigman, 1998). Importantly, however, children vary widely in the timing, complexity, and trajectory of play development, which highlights the need to understand the factors that predict individual differences in play outcomes (Panganiban & Kasari, 2022).

This variability is particularly pronounced among autistic children, who often show significant delays in symbolic play. While some autistic children demonstrate functional play skills, symbolic and pretend play behaviors are often absent, or limited in range during the preschool years (Rutherford et al., 2007). At the same time, there is a substantial heterogeneity in play development within this population. Differences in joint attention, imitation, language abilities, cognitive functioning, and restricted interests may contribute to wide variability in the form and the sophistication of play skills (Chang et al., 2024). Given the strong developmental links between symbolic play, joint engagement, and social communication, understanding which child characteristics predict play outcomes and whether parents can facilitate these changes with participation in early interventions is critical. Play skills have also been shown to predict later peer relationships and broader social functioning (Freeman et al., 2015, Sigman, 1998), suggesting that early differences in play development may have cascading developmental consequences.

Despite the developmental importance of play, relatively few early intervention programs explicitly target play development in young autistic children (e.g., Chang et al., 2024). Many early intervention models focus on social communication goals within play-based contexts, often assuming that improvements in play will occur as a downstream effect of increased social engagement. However, emerging evidence suggests that without explicit and targeted support, higher forms of play may not fully develop or generalize, particularly for autistic children whose social communication profiles include limited joint attention (Panganiban & Kasari, 2022). These patterns highlight the importance of examining not only whether play-based interventions are effective for improving play skills, but for whom they are most effective and under what conditions.

### 1.1. Parent-Mediated Interventions

Parent-mediated interventions have become an increasingly common and promising approach to supporting young autistic children (Deniz et al., 2024; Nevill et al., 2018). These programs provide parents with psychoeducation, training, and coaching by trained professionals, positioning parents as the primary agents of change within their child’s everyday environment. When parents are supported in implementing intervention strategies, research has demonstrated improvements in children’s social communication and increases in caregiver responsiveness and interaction quality (Brian et al., 2022; Shire et al., 2022). Nevertheless, outcomes within parent-mediated interventions are variable, suggesting that child- and family-level factors may play a critical role in shaping responsiveness and developmental gains.

Notably, the majority of parent-mediated intervention research has been conducted with higher income and educated families who experience fewer structural barriers to accessing services (Dijkstra-de Neijs et al., 2023). Families from under-resourced, culturally and ethnically diverse, or marginalized communities remain underrepresented in research and disproportionally underserved in practice (Carr & Lord, 2016; Pierce et al., 2014; West et al., 2016). These families often encounter compounding barriers, including limited financial resources, language differences, insurance coverage, transportation challenges, demanding work schedules, and competing caregiver responsibilities (Gulsrud et al., 2021; Liu et al., 2023; K. A. Smith et al., 2020; Stahmer et al., 2019). Such barriers can limit access to research-supported early interventions that are traditionally carried out in clinics, or available to only those who live close to university locations (Carr & Lord, 2016; Sterrett et al., 2023). As a result, examining predictors of early intervention outcomes with a diverse set of families who may be from under-resourced contexts casts a wider net for understanding our current evidence base.

Home-based, parent-mediated interventions may help with the inclusion of a diverse group of families, especially when the focus is on embedding intervention strategies within families’ natural environments and daily routines, such as mealtime, grooming, and play (Woods et al., 2004). These routines provide frequent and meaningful opportunities for parent–child interaction and skill development. However, although home-based models are often designed to promote social communication, less attention has been devoted to directly supporting the development and generalization of play skills within everyday contexts. Moreover, prior studies have frequently relied on parent report measures or observations conducted during intervention sessions, which may not capture how consistently or effectively strategies are implemented during daily routines. Understanding how play-related outcomes unfold in naturalistic home environments (outside of therapeutic sessions) is particularly important for evaluating the ecological validity and sustainability of intervention effects.

### 1.2. Predictors of Intervention Outcomes

At the child level, developmental characteristics such as baseline play skills, language abilities, joint attention, imitation, and cognitive functioning have been shown to influence responsiveness to early intervention, including gains in play and social communication (Chang et al., 2024; Panganiban & Kasari, 2022; T. Smith et al., 2015). Children with advanced functional or emerging symbolic play skills may show faster growth, whereas children with limited imitation or restricted interests may require more explicit and intensive support to benefit from play-focused approaches (Panganiban & Kasari, 2022; Panganiban et al., 2025). At the family level, factors such as maternal age and education have also been associated with variability in intervention outcomes (Itzchak & Zachor, 2011). Examining child- and family-level predictors together allows for a more nuanced understanding of variability in play outcomes and can inform the tailoring of interventions to better align with the strengths, needs, and contexts of families.

### 1.3. Current Study

The current study aims to examine child- and family-level predictors of play outcomes within a home-based, parent-mediated intervention for young autistic children in low-income contexts.

## 2. Methods

### 2.1. Participants

The current study is a secondary analysis of data from a larger randomized controlled trial focusing on early intervention for low-income and under-resourced populations (Kasari et al., 2014). The study recruited families from five sites across the country. Families were defined as low-resourced by one of two options: (1) low income as indicated by the US Department of Housing and Urban Development, or (2) one of the following factors: mother held a high school diploma or lower; currently unemployed; or receiving government assistance. Children were between 2 and 5 years of age, had a mental age above 12 months, and had a clinical diagnosis of ASD using the Autism Diagnostic Observation Scale (ADOS) confirmed by independent assessors. Children were excluded from the study if they had known genetic co-morbidities (e.g., Down syndrome, Fragile X).

The original study had a sample of 112 caregiver–child dyads. Of the original 112 dyads, 97 dyads had videotapes of home observations at both entry and exit. The current study included only the 97 dyads with complete home observation data. The children’s average age was 42.3 months (*SD* = 10.1 months). Their average mental age was 24.72 months (*SD* = 11.77 months). The families were of diverse backgrounds: 34% White, 26% African American, 14% Hispanic, 15% Multiethnic/Other, and 8% Asian. The majority of the mothers (79%) have some college or a college degree. Demographic characteristics are presented in Table 1 and Table 2.

### 2.2. Intervention

Caregivers were randomized to two different treatment groups in the study: Caregiver-mediated (CMM) or Caregiver Education (CEM). Caregivers in the CMM group received active coaching from trained interventionists to play with their children in their homes. The intervention involved two 1-hour sessions per week for 12 weeks. The interventionists followed two manualized protocols: (1) establishing home routines that involve play and everyday activities, and (2) actively coaching caregivers to implement strategies using the JASPER intervention, a play-based naturalistic developmental behavioral intervention. The active coaching session included strategies such as setting up the learning environment with developmentally appropriate materials, establishing and expanding developmentally appropriate home routines, programming opportunities for social communication skills, and modeling developmentally appropriate language.

Fidelity was rated during a 10-minute caregiver–child interaction by blinded, independent coders. Five broad domains, consisting of various specific strategies, were coded for JASPER parent strategy use. These included the environment, imitation, prompting, communication, and engagement. Each individual strategy was coded for each of the two-minute intervals within the ten-minute interaction. Overall, total caregiver fidelity averaged 76% with a range from 41% to 99%.

Caregivers in the CEM group received small group-based treatment focusing on supporting their children in behavioral management and communication. The group consisted only of caregivers and was led by a trained interventionist in centralized neighborhood locations (e.g., homes, community centers, clinics, and schools). The caregivers attended two-hour group sessions each week for 12 weeks. Caregiver fidelity averaged 97% with a range of 83% to 100%.

### 2.3. Measures

*The Autism Diagnostic Observation Schedule* (ADOS; Lord et al., 2012). The ADOS is a semi-structured, standardized assessment used to evaluate communication, social interaction, and play. It is designed to identify clinical features consistent with autism spectrum disorder. Module 1, Module 2, or Module 3 was administered based on the child’s developmental and expressive language levels. The ADOS yields algorithm scores across two domains, language/communication and reciprocal social interaction, which are used to determine diagnostic classification. Additional domains, including play and imagination/creativity, stereotyped behaviors, and restricted interests, contribute to clinical interpretation but are not included in the diagnostic algorithm. All children were assessed at baseline and met ADOS algorithm criteria for autism or autism spectrum disorder.

*The Mullen Scales of Early Learning* (MSEL; Mullen, 1995). The MSEL is a standardized developmental assessment with strong construct, convergent, and divergent validity for use with both typically developing children and autistic children (Bishop et al., 2011; Swineford et al., 2015). The MSEL assesses functioning across four domains: visual reception, fine motor, receptive language, and expressive language. Children’s mental age was calculated by averaging the age-equivalent scores across these four domains.

*Early Social-Communication Scales* (ESCS; Mundy et al., 2003). The ESCS is a 30 min semi-structured assessment used to measure children’s joint attention and behavior regulation skills. The type and frequency of spontaneous initiations of joint attention skills (IJA) and behavior requests (IBR), including eye contact, point, give, show, and language, were coded. Independent coders who were blind to treatment conditions and timepoints coded the ESCS. Inter-rater reliability among 8 independent coders blind to group status was ICC = 0.80.

*Home Observation.* The home observation consisted of a 60 min videotaped session used to capture naturalistic home routines and activities in which the child and caregiver typically engaged together. At the start of the session, a videographer who was blind to the families’ treatment group assignments read a standardized script instructing caregivers to interact with their child as they normally would during everyday activities. Caregivers were provided with five categories of activities to guide the session: play (e.g., blocks, barn with farm animals), meals and snacks (e.g., meal preparation, eating), caregiving routines (e.g., washing hands, dressing), book sharing, and family chores (e.g., sweeping, folding laundry). The caregivers were encouraged to engage in any combination of activities they typically did with their children.

The home observations were coded into two primary activity categories: play and non-play activities (including meals, caregiving routines, book sharing, and family chores). For families who engaged in play, the quality of play was further coded as either functional or symbolic. Functional play included sensory activities (e.g., bubbles), cause-and-effect toy play (e.g., pop-up toy), and constructive play using building materials (e.g., blocks). Symbolic play included the substitution of objects, the use of figures (e.g., dolls engaging in activities) and pretend play (e.g., a child pretends to be a superhero). The inter-rater reliability was ICC = 0.89.

### 2.4. Data Analyses

Descriptive statistics and bivariate tests were utilized to describe and evaluate child and caregiver characteristics by intervention groups. McNemar’s tests were conducted to examine the types of home activities caregivers engaged with their children from entry to exit (i.e., paired proportions). Only 3% (n = 1) of dyads demonstrated symbolic play at entry; hence, changes in odds of symbolic play cannot be evaluated, as there was no variability in symbolic play at entry. Therefore, logistic regression was utilized to evaluate potential baseline predictors of symbolic play (binary: symbolic vs. functional) at exit while controlling for intervention effects. All predictors are evaluated individually to avoid collinearity, as child predictors were coded from the same assessments (i.e., joint attention and behavioral requests are from the ESCS).

## 3. Results

### 3.1. Child Characteristics

There were no significant differences in age, gender, cognitive abilities, and language skills between the two groups (CMM and CEM). Comparisons in demographic variables between groups were evaluated using ANOVA (analysis of variance) for continuous variables, and the chi-square test was used to evaluate the distribution of categorical variables between groups.

### 3.2. Home Observations

*Home Activities and Routines*. There were no group differences in home activities between the two treatment groups from entry to exit (*p* = 0.125–0.999). The two treatment groups were then combined to examine the types of activities families chose to engage in with their children. Ninety-six percent of the families chose play as a home activity to engage in with their children (see Table 3).

*Time Spent in Play Activities.* An independent t-test was conducted to examine the difference in time spent in play activities between the two treatment groups, CEM and CMM. There were no differences between the time spent by caregivers playing with their children from entry to exit between the two groups (*t*(95) = −0.48, *p* = 0.633) (See Table 4).

*Functional and Symbolic Play.* Ninety-seven percent of the children engaged in functional play at entry. By exit, approximately only 82% of the children were engaged in functional play, with 18% of the children playing symbolically at exit. Children in the CMM group had significantly greater odds of having symbolic play at exit compared to children in the CEM group (OR = 3.71, *Z* = 2.03, *p* = 0.042).

*Predictors of Symbolic Play.* Children’s baseline initiations of joint attention skills (OR = 1.03, *Z* = 2.14, *p* = 0.03), initiations of behavioral requests (OR = 1.04, *Z* = 2.26, *p* = 0.024) and mental age (OR = 1.07, *Z* = 3.04, *p* = 0.002) predicted increased odds of having symbolic play. RJA (OR = 1.01, *Z* = 0.16, *p* = 0.873) and RBR (OR = 0.98, *Z* = −0.78, *p* = 0.449) did not predict changes in the odds of having symbolic play. Family characteristics such as income (OR = 1.05, *Z* = 0.808, *p* = 0.419) and education (OR = 1.64, *Z* = 0.90, *p* = 0.368) also did not predict to children’s symbolic play at exit.

## 4. Discussion

The study identified child-level predictors of play outcomes within a home-based, parent-mediated intervention for young autistic children. Specifically, baseline initiations of joint attention, initiations of behavior requests, and mental age were all significant predictors of change in symbolic play, whereas no family-level predictors, including household income and maternal education, were significantly associated with play outcomes. These findings highlight the importance of understanding which child characteristics influence responsiveness to play-based interventions and the potential for targeted, individualized approaches that account for developmental readiness.

Initiations of joint attention and initiations of behavioral requests were significant predictors of symbolic play outcomes, highlighting the central role of child-initiated social communication in supporting play development. Initiations of joint attention reflect children’s ability to coordinate their attention with a social partner and an object, while initiations of behavior requests indicate an understanding of intentional communication to achieve a goal (e.g., obtaining an object). These skills likely facilitate engagement with caregivers during play and create opportunities for shared meaning-making, which is essential for the emergence and elaboration of higher levels of play. The findings suggest that the children who demonstrate more initiations of social communication and interactions may benefit more quickly from play-based intervention, whereas children with fewer initiations may require more explicit support from their adult social partners to promote engagement and reciprocity within play routines. This finding aligns with previous studies that have examined play in autistic children (Chang et al., 2018; Panganiban & Kasari, 2022).

Notably, no family-level predictors were significantly associated with symbolic play outcomes. The finding is particularly important given the study’s focus on under-resourced families, as it challenges the assumption that socioeconomic factors, such as income or maternal education, necessarily impact intervention effectiveness. The absence of significant family-level predictors suggests that when parents are provided with structured, developmentally informed, evidence-based strategies and support within their natural environments, children’s developmental characteristics may play a more central role in shaping outcomes than socioeconomic risk. This pattern provides encouraging evidence that well-designed, home-based parent-mediated interventions may serve as a buffer against structural inequities that have historically limited access to and benefits from early intervention services.

Beyond identifying predictors of change, the current study also contributes to the literature by demonstrating the value of examining play outcomes within families’ everyday routines. By observing parent–child interactions during natural home activities outside of therapy sessions, the study provides insight into how intervention-related gains in symbolic play generalize beyond structured sessions. Symbolic play is a foundational developmental skill linked to later social engagement, school readiness, and peer relationships (Freeman et al., 2015; Sigman, 1998), yet it is often underemphasized as a primary outcome in early intervention research. The finding suggests that play-based intervention, such as JASPER, can support meaningful changes in children’s spontaneous play behavior in real-world contexts, particularly for children who enter the intervention with certain developmental strengths.

The study also has some limitations. Play was coded using broad categories, functional and symbolic, which may not fully capture the nuances in children’s play skills. This approach does not account for variations in frequency and complexity, or the specific subtypes of functional and symbolic play skills that children may demonstrate within naturalistic contexts. Future research would benefit from using a more fine-grained coding framework that captures these distinctions to provide a more comprehensive understanding of how play develops in response to intervention.

Importantly, identifying predictors of play outcomes also has direct implications for intervention design and refinement. Understanding which children are more likely to benefit from play-based intervention can inform decisions about when additional supports and adaptations may be needed (Panganiban & Kasari, 2022; Panganiban et al., 2025). For example, children with fewer initiations of joint attention and behavioral requests may benefit from early interventions that specifically target these skills whether through naturalistic developmental strategies or direct instruction (e.g., Kasari et al., 2025). Future research can move beyond describing average treatment effects and inform more precise, responsive, and adaptive intervention models.

Lastly, the findings have implications for health equity and service delivery. By demonstrating that child-level developmental characteristics, rather than family socioeconomic indicators, predict symbolic play outcomes within an under-resourced sample, the study supports the feasibility and potential effectiveness of home-based, parent-mediated interventions in marginalized communities. Shifting toward flexible, community-grounded service delivery models that emphasize caregiver coaching within daily routines may help reduce disparities in access, participation, and long-term sustainability. Identifying predictors of response within these contexts is a critical step toward ensuring that early intervention services are not only effective, but also equitable and responsive to the diverse needs of families.

## 5. Conclusions

The study highlighted the importance of identifying predictors of play outcomes to inform early intervention practices for young autistic children. The results demonstrated that child-level developmental characteristics were associated with gain in symbolic play, whereas family-level socioeconomic factors were not. This suggests that when parents are adequately supported through structured, home-based coaching, children’s developmental profiles may be more influential than structural disadvantages in shaping the response to intervention. These findings support the use of accessible, parent-mediated, play-based interventions as a means of reducing disparities in early intervention outcomes and highlight the importance of tailoring supports based on child characteristics rather than assumptions about family resources. Future studies should examine targeted, individualized approaches to early intervention applied to diverse groups of participants to further expand the early intervention evidence base.

## Figures and Tables

**Table 1:** Child characteristics, *n* (%).

	CEM, *n* = 52	CMM, *n* = 60
Chronological age, mo: mean (*SD*)	42.8 (10.21)	41.9 (10.0)
Gender		
Male	43 (82.7%)	50 (83.3%)
Female	9 (17.3%)	10 (16.6%)
Race/ethnicity		
White	16 (30.8%)	23 (38.3%)
Hispanic	7 (13.5%)	9 (15.0%)
African American	18 (34.6%)	13 (21.7%)
Asian	4 (7.7%)	5 (8.3%)
Multi-ethnic/other	7 (13.5%)	10 (16.7%)
Language child hears most at home		
Non-English	5 (9.6%)	10 (16.6%)
Receives other early intervention services		
No	10 (19.23%)	10 (16.67%)
Yes	42 (80.77%)	48 (80.0%)
Missing	0 (0%)	2 (3.33%)
Mullen age equivalency, mo: mean (*SD*)		
Mental age	26.3 (11.8)	23.6 (11.6)
Receptive language	23.3 (13.2)	22.1 (14.4)
Expressive language	24.1 (13.5)	20.1 (12.6)
Fine motor	29.1 (10.8)	25.5 (9.3)
Gross motor	28.8 (12.2)	26.4 (12.7)
ESCS		
Joint attention skills	14.65 (18.01)	9.39 (12.68/8)
Behavioral request	16.96 (12.25)	16.75 (14.45)
Responding to joint attention	21.69 (5.08)	19.66 (6.37)
Responding to behavioral request	24.08 (11.53)	22.14 (10.42)
ADOS severity score: mean (*SD*)		
Module 1 (1 to 10)	7.53 (1.8)	7.6 (2.3)
Module 2 (1 to 10)	6.3 (1.1)	6.4 (1.9)
Module 3 (1 to 10)	6.0 (0)	7.7 (0.6)

Note: Caregiver-mediated (CMM) or Caregiver Education (CEM).

**Table 2:** Caregiver characteristics.

	CEM, *n* = 52	CMM, *n* = 60
Maternal education		
<12th grade	3 (5.8)	4 (6.7)
High school diploma or GED	8 (15.4)	9 (15.0)
Some college/college degree	29 (55.8)	36 (60)
Graduate work/graduate degree	12 (23)	9 (15)
Unknown	0 (0)	2 (3.3)
Low income		
No	19 (36.5)	19 (31.7)
Yes	31 (59.6)	38 (63.3)
Unknown	2 (3.9)	3 (5.0)
Receives Medicaid	16 (30.8)	23 (38.3)
Receives any assistance	27 (51.9)	35 (58.3)

**Table 3:** Number of dyads per type of activity at entry and exit.

	CMM		CEM		Combined	
	Entry	Exit	Entry	Exit	Entry	Exit
Play	96%	100%	96%	100%	96%	100%
Meal	38%	29%	38%	56%	38%	41%
Book Reading	44%	37%	44%	56%	44%	45%
Caregiving	44%	23%	22%	31%	34%	27%
Family Chores	10%	17%	13%	18%	11%	18%

**Table 4:** Time spent in play (minutes).

Mean (SD)	CEM	CMM
Baseline	29.6 (15.6)	29.5 (14.0)
Exit	27.5 (11.1)	28.9 (11.0)

## Data Availability

The data supporting the conclusions of this article may be made available by the authors on request.
